# Inferior Oblique Muscle Weakening: Is It Possible to Quantify Its Effects on Horizontal Deviations?

**DOI:** 10.1155/2012/813085

**Published:** 2012-12-16

**Authors:** Hande Taylan Sekeroglu, Ozlem Dikmetas, Ali Sefik Sanac, Emin Cumhur Sener, Umut Arslan

**Affiliations:** ^1^Department of Ophthalmology, Hacettepe University School of Medicine, 06100 Ankara, Turkey; ^2^Department of Biostatistics, Hacettepe University School of Medicine, 06100 Ankara, Turkey

## Abstract

*Objective*. To evaluate and quantify the effect of inferior oblique muscle weakening on horizontal deviations. *Methods*. The medical files of patients who had undergone an inferior oblique weakening as a single procedure were all reviewed. The main measures were the type of inferior oblique overaction (IOOA), pre- and postoperative amount of IOOA, and horizontal deviations in primary position. *Results*. The study was conducted with 66 patients (30 males, 36 females). The median age was 11 years (1–49). Of the 66 patients, 30 (45.5%) had primary and 36 (54.5%) had secondary IOOA. The most common procedure was inferior oblique anteriorization in 32 patients (48.5%). The mean postoperative horizontal and vertical deviations and the amount of IOOA were decreased postoperatively (*p* = 0.001 for all). The median amount of correction of horizontal near and distance deviations was 4Δ (0–20). The preoperative amount of IOOA, the presence of fourth nerve palsy, and the type of the weakening procedure had no significant effect on the amount of correction of horizontal deviations. *Conclusion*. The inferior oblique weakening procedures have secondary effects and warrant reduction of horizontal deviations in varying degrees. This should be borne in mind in planning a simultaneous horizontal muscle surgery and setting the surgical amount.

## 1. Introduction

Inferior oblique muscle overaction (IOOA) may be primary or secondary. The former occurs in 72% of congenital esotropia, 34% of accommodative esotropia, and 32% of intermittent exotropia [1]. The most common cause of secondary IOOA is fourth nerve palsy. 

A variety of procedures have been performed including recession, hang-back recession, myotomy, myectomy, anterior transposition, nasal transposition, denervation, and muscle fixation [2–7]. However, these surgeries may have effect not only on vertical deviations but also on horizontal deviations in varying degrees. There is a knowledge that the weakening of the inferior oblique muscles causes an esodeviation about 5–10 PD, and the weakening of the superior oblique muscles causes an esodeviation about 10–15 PD [8]. There are a number of previous studies that were designed to provide comparative data on inferior oblique muscle weakening surgeries' effects on vertical deviations [4, 7, 9–11]. According to this data, there is a possibility that inferior oblique weakening may affect by itself horizontal alignment in primary position, may influence plans and numbers of horizontal muscle surgeries, and furthermore it may be enough in a particular amount of deviations to provide horizontal alignment without need for additional horizontal surgeries. The intention of the present study was to evaluate the effect of inferior oblique muscle weakening on horizontal deviation in primary position and to empirically determine the relationship between the type of the procedure and the amount of the effect if there was any and to be able to predict the horizontal aligning effect.

## 2. Materials and Methods

This retrospective study was conducted in Hacettepe University Medical School, Ophthalmology Department, Pediatric Ophthalmology and Strabismus Section. The study protocol was approved by the institutional review board. The procedures conformed with the tenets of the Declaration of Helsinki. The consecutive patients who had undergone the inferior oblique muscle weakening surgery (tenotomy, recession, or anteriorization) as a sole procedure between 2001 and 2012 and who had postoperative control examination and whose orthoptic evaluation could be performed completely were enrolled in the study. The exclusion criteria were described in Table 1. 

The amounts of IOOA were classified as +1 to +4. Diagnosis was based on evaluation of ocular misalignment in 9 diagnostic gaze positions, and Hess Lee screen tests if applicable. Ocular misalignment was measured with the prism cover test or Krimsky. The retrospective data collected included the diagnosis at presentation, pre- and postoperative horizontal and vertical deviations in primary position, presence or absence of fourth nerve palsy, type of the inferior oblique weakening surgery including tenotomy, recession, or anteriorization, previous surgery of horizontal muscles, pre- and postoperative degree of IOOA, and the effect of inferior oblique muscle weakening intervention on the amount of horizontal near and distance deviations in primary position. 

The insertion of the inferior oblique muscle was placed at the temporal border of the inferior rectus muscle in anteriorization and generally 2 mm posterior to the lateral of inferior rectus muscle in the recession procedure. The type of surgery was determined according to the surgeon's preference.

The main measures were the type of IOOA, pre- and postoperative amount of IOOA, and horizontal deviations in primary position. 

Postoperatively, the subjects were examined at three months after surgery. 

Data were analyzed using SPSS 15.0 for Windows (SPSS Inc., Chicago, IL). For normal distribution of quantitative data, independent samples *t*-test was used for comparison of two independent groups. Mann-Whitney *U* test was used for abnormal distribution of quantitative data and Wilcoxon signed-rank test was used to compare quantitative variables in the matched sample. Categorical variables were analyzed by Yate's correction chi-square test. Arithmetic mean, standard deviation, median, and range were given as descriptive statistics for quantitative data. Qualitative data are summarized using frequency and percentages. Results were accepted as statistically significant when *p* was <0.05. 

## 3. Results

Sixty-six patients (30 males and 36 females) with a median age of 11 years (1–49) were enrolled in the study. The median age of males was 14 years (2–49) and the median age of females was 6.5 years (1–39) (*p* = 0.046). 

Of the 66 patients, 36 (54.5%) had secondary IOOA due to fourth nerve palsy and 30 (45.5%) had primary IOOA. The most commonly performed surgery was inferior oblique anteriorization in 48.5% (32 patients), followed by inferior oblique tenotomy in 43.9% (29 patients), and recession in 7.6% (5 patients). Of the 66 patients, 39 (59.1%) underwent unilateral and 27 (40.9%) bilateral surgery.

Of the 66 patients, 9 (13.6%) had undergone previous horizontal muscle surgeries. 

The median amount of IOOA in patients reduced from +3 (1–4) to 0 (0–2) in patients with secondary IOOA and from +3 (2–4) to +1 (0–2) in patients with primary IOOA (*p* = 0.001 for both). Of the 66 patients, 43 (65.1%) had no residual IOOA at the postoperative visit. 

The amount of horizontal near, distance, and vertical deviations was significantly decreased (*p* = 0.001 for all, Table 2). 

The amount of correction in deviations was shown in Table 3. There was 4Δ (0–20) esoshift in horizontal near deviation and 4Δ (0–20) esoshift for horizontal distance deviation. Furthermore, there was no difference of the median amount of correction between cases with primary and secondary IOOA in terms of horizontal deviations. When postoperative correction of horizontal near and distance deviations was considered, the amount of preoperative level of IOOA (*p* = 0.081 and *p* = 0.127), the type of the inferior oblique weakening procedure (*p* = 1.000 and *p* = 0.734), the presence of previous horizontal muscle surgery (*p* = 0.977 and *p* = 0.568), the presence of fourth nerve palsy (*p* = 0.207 and *p* = 0.630), and laterality of the surgery (*p* = 0.272 and *p* = 1.000) had no significant effect. The sole factor affecting the amount of correction of horizontal near deviations was preoperative amount of horizontal distance deviations (*p* = 0.027). The preoperative amount of horizontal near deviation and vertical deviation had no effect (*p* = 0.176 and *p* = 0.957). There was no intra and postoperative complication during followup.

## 4. Discussion

Weakening surgery for IOOA either primary or secondary includes recession, disinsertion and myectomy, tenotomy, marginal myotomy, and anteriorization. The aim of all these surgeries is to release IOOA and reduce related vertical deviations. The inferior oblique muscle is an abductor and its weakening might induce esoshift. In light of this basic information, in spite of the variability of the results, our starting point was the following question in the present study: could we have an estimation about the effect of the inferior oblique muscle weakening procedure on the magnitude of horizontal deviations and if it was possible to quantify it because if we can predict its effect on horizontal alignment, we can have the possibility to adjust our numbers for horizontal surgeries. There is few data in the literature about horizontal aligning effect of oblique muscle surgeries. 

Souza-Dias [12] investigated the effect of superior oblique muscle weakening on horizontal alignment in primary position and found esoshift in three patients and exo-shift in four patients whereas the horizontal alignment in primary position was not affected in five patients and calculated that this procedure caused 2.25Δ exo-shift. In the present study, we found a median amount of 4Δ esoshift in horizontal near and distance measurements in primary position regardless of the type of surgery for inferior oblique muscle. 

Stager et al. [13] showed that unilateral anterior and nasal transposition of inferior oblique muscle corrected 13Δ vertical deviation in primary position in a sample of 20 patients with diagnosis including IOOA, superior oblique palsy, absent superior oblique muscles, antielevation syndrome, and Duane syndrome, but they noticed that this particular procedure could induce exotropia in primary position. 

All aforementioned studies had largely examined and analyzed the vertical effects of inferior oblique muscle weakening surgeries and related possible complications. However, their effects on horizontal deviations have not been described in detail. In this paper, we described our findings in a series of patients who underwent weakening procedure as a sole intervention and whose horizontal deviations were believed to have been ameliorated and found that weakening of inferior oblique muscle had significant effect on horizontal deviations. Although recession, anteriorization, and disinsertion were thought to have different effects on the horizontal alignment on primary position in regard of different axis of action, we did not found any difference between different types of surgery in terms of horizontal deviation correcting effect. The present data confirmed in brief that weakening of inferior oblique muscle may act as a horizontal deviation correcting procedure and may reduce the amount of horizontal misalignment.

It was not clear that the larger the preoperative horizontal deviations, the greater the amount of correction was obtained postoperatively. We found that the postoperative reduction of horizontal deviation was not related with preoperative amount of horizontal near and vertical deviations but it was influenced by preoperative amount of horizontal distance deviation. However, the reason for this relationship remained unexplained. The weakening caused an esoshift in primary position, which may be an expectable result in regard of inferior oblique muscle's functions. 

This study needs to be viewed in light of the following limitations: small number of cases, lack of control group, retrospective nature, and short-term followup. The presence of a previous horizontal muscle surgery may have an indirect long-term effect and may prohibit the evaluation of the pure effect of inferior oblique muscle weakening on horizontal deviations. Furthermore, the binocular status of the patients was not mentioned and described in the study and it may be a main topic for another study. However, weakening of inferior oblique muscle had shown promising results in correction of horizontal deviations. The question arises whether the effect of weakening on horizontal deviation may be predicted and quantified. This presumption would not be possible and realistic, because scaling of the surgical amount is always challenging for selected strabismus surgeries, in regard of the difficulty of the standardization of all steps; so the results of surgery may be surgeon specific and not generalizable, even not relevant because the median amount of diopter change was small (4Δ) and may be affected from the measurement errors and the diversity of techniques of each surgeon.

In conclusion, when planning and performing a strabismus surgery which targets weakening of the inferior oblique muscle, it is important to have a thorough knowledge of its possible effects on horizontal deviations. Even if it is performed as a sole procedure, inferior oblique weakening, whatever the type of procedure is, may affect the horizontal alignment in primary position and may influence the surgical numbers for future horizontal muscle surgeries in so much that the numbers may need to be decreased particularly for exodeviations. This influence should be borne in mind.

## Figures and Tables

**Table 1:** The exclusion criteria of the study.

Exclusion criteria	
(1) Restrictive strabismus	
(2) Uncooperation during measurements	
(3) Combined surgery of horizontal and/or vertical muscles aside from the inferior oblique muscle weakening procedure	
(4) History of trauma	
(5) Neurological, genetic, or craniofacial abnormalities	

**Table 2:** The change of deviations after surgery in patients with inferior oblique overaction.

	Type of IOOA							
Deviations	Primary		*Z*	p*	Secondary		*Z*	p*
	Preoperative Median (min−max)Δ	Postoperative Median (min−max)Δ			Preoperative Median (min−max)Δ	Postoperative Median (min−max)Δ		
Horizontal near	14 (2–50)	10 (0–50)	−**3.425**		9 (0–50)	0 (0–50)	−**4.471**	
Horizontal distance	12 (2–55)	10 (0–55)	−**3.417**	**0.001**	7 (0–55)	0 (0–50)	−**4.043**	**0.001**
Vertical	9 (0–40)	0 (0–20)	**−3.825**		12 (4–40)	0 (0–20)	**−5.019**	

*Wilcoxon signed-rank test.

**Table 3:** The amount of change in deviations after the inferior oblique muscle weakening procedures.

Deviations	Type of IOOA		*Z *	p*
	Primary Median (min−max)Δ	Secondary Median (min−max)Δ		
Horizontal near	2 (0–14)	4 (0–25)	−1.263	0.207
Horizontal distance	2 (0–14)	4 (0–30)	−0.482	0.630
Vertical	4 (0–30)	10 (0–30)	−2.825	**0.005**

*Mann-Whitney test.
